# Sensitivity of osteosarcoma cells to HDAC inhibitor AR-42 mediated apoptosis

**DOI:** 10.1186/s12885-017-3046-6

**Published:** 2017-01-21

**Authors:** Sridhar Murahari, Aimee L. Jalkanen, Samuel K. Kulp, Ching-Shih Chen, Jaime F. Modiano, Cheryl A. London, William C. Kisseberth

**Affiliations:** 1 Department of Veterinary Clinical Sciences, College of Veterinary Medicine, The Ohio State University, Columbus, OH 43210 USA; 2 Department of Veterinary Biosciences, College of Veterinary Medicine, The Ohio State University, Columbus, OH 43210 USA; 3 Division of Medicinal Chemistry and Pharmacognosy, College of Pharmacy, The Ohio State University, Columbus, OH 43210 USA; 4 Department of Veterinary Clinical Sciences, College of Veterinary Medicine, University of Minnesota, St. Paul, MN 55108 USA; 5 Masonic Cancer Center, University of Minnesota, Minneapolis, MN 55455 USA; 6 Current address: Department of Microbiology, Immunology and Pathology, Colorado State University, Fort Collins, CO 80523 USA

**Keywords:** Apoptosis, AR-42, Dog, Histone deacetylase inhibitor, Osteosarcoma

## Abstract

**Background:**

Osteosarcoma (OS) is the most common primary bone tumor in both humans and dogs and is the second leading cause of cancer related deaths in children and young adults. Limb sparing surgery along with chemotherapy has been the mainstay of treatment for OS. Many patients are not cured with current therapies, presenting a real need for developing new treatments. Histone deacetylase (HDAC) inhibitors are a promising new class of anticancer agents. In this study, we investigated the activity of the novel HDAC inhibitor AR-42 in a panel of human and canine OS cell lines.

**Methods:**

The effect of AR-42 and suberoylanilide hydroxamic acid (SAHA) alone or in combination with doxorubicin on OS cell viability was assessed. Induction of histone acetylation after HDAC inhibitor treatment was confirmed by Western blotting. Drug-induced apoptosis was analyzed by FACS. Apoptosis was assessed further by measuring caspase 3/7 enzymatic activity, nucleosome fragmentation, and caspase cleavage. Effects on Akt signaling were demonstrated by assessing phosphorylation of Akt and downstream signaling molecules.

**Results:**

AR-42 was a potent inhibitor of cell viability and induced a greater apoptotic response compared to SAHA when used at the same concentrations. Normal osteoblasts were much less sensitive. The combination of AR-42 with doxorubicin resulted in a potent inhibition of cell viability and apparent synergistic effect. Furthermore, we showed that AR-42 and SAHA induced cell death via the activation of the intrinsic mitochondrial pathway through activation of caspase 3/7. This potent apoptotic activity was associated with the greater ability of AR-42 to downregulate survival signaling through Akt.

**Conclusions:**

These results confirm that AR-42 is a potent inhibitor of HDAC activity and demonstrates its ability to significantly inhibit cell survival through its pleiotropic effects in both canine and human OS cells and suggests that spontaneous OS in pet dogs may be a useful large animal model for preclinical evaluation of HDAC inhibitors. HDAC inhibition in combination with standard doxorubicin treatment offers promising potential for chemotherapeutic intervention in both canine and human OS.

## Background

Osteosarcoma (OS) is the most common primary malignant bone tumor in humans, affecting primarily adolescents. Metastasis occurs frequently, with the lungs being the most common metastatic site. Metastases occur in greater than 80% of affected individuals treated with surgery alone. Despite aggressive treatment, about a third of affected patients die of their disease. Current treatment with neoadjuvant chemotherapy followed by surgical resection and additional adjuvant chemotherapy results in a 5-year-survival rate of 60–70% for people with non-metastatic osteosarcoma treated with combinations of methotrexate, cisplatin, doxorubicin and ifosfamide [1]. Survival rates have improved little over the past 30 years, necessitating the development of new therapeutic approaches.

Spontaneous OS in the dog is an excellent large animal model for the human disease. Interestingly, OS is also the most common primary bone tumor in dogs, occurring in the canine population with an estimated incidence of at least 13.9/100,000 [2] compared to the human incidence of 1.02/100,000 [3]. The pathobiological, clinical, and molecular characteristics of the disease in humans and dogs are quite similar. Dysregulated expression of ezrin, Met, STAT3, Her2/Neu, overlapping transcriptional profiles, and extreme genomic instability are among the shared molecular characteristics in OS in the two species. As in people, OS in dogs is an invasive rapidly growing cancer with a high metastatic potential, primarily to lung and bone. The median survival time for affected dogs treated with amputation or limb sparing surgery and chemotherapy with a platinum drug or doxorubicin is 9–12 months, with most dogs eventually dying from metastatic disease. As with people, survival times have not improved appreciably since incorporation of the adjuvant chemotherapy [4]. Clearly, new treatment approaches are needed to improve outcomes for OS in both humans and dogs.

Histone acetylation plays a significant role in transcriptional regulation by altering the structure of chromatin. Acetylation of core histones is regulated by the opposing activities of histone acetyl transferases (HATs) and histone deacetylases (HDACs) that alter the transcriptional status of the chromatin [5, 6]. HATs catalyze transfer of acetyl groups to NH2-terminal lysine residues in histones that results in an open chromatin conformation and transcriptional activation by increasing the accessibility of transcriptional machinery. In contrast, recruitment of the HDAC complex results in chromatin condensation and transcriptional repression [6]. Thus, aberrant recruitment of the HDAC complex represses the transcription of specific tumor suppressor genes resulting in aberrant regulation of gene expression [7]. Several lines of evidence indicate that altered HAT and HDAC activities are mechanistically linked to pathogenesis of a variety of cancers as well as other diseases [7]. Therefore, HATs and HDACs are promising targets for therapeutic interventions as they are directed to reversing the aberrant epigenetic modifications in neoplastic cells in contrast to genetic mutations that are irreversible [8]

HDAC inhibitors represent a class of anticancer agents that modulate the transcription of target genes by regulating the access of transcription factors and RNA polymerases to the promoter regions. In this context, many HDAC inhibitors have been developed and extensively investigated for their potential for anticancer treatment, resulting in the FDA approval of Zolinza (vorinostat), Istodax (romidepsin) and Beleodaq (belinostat) for the treatment of cutaneous and peripheral T cell lymphoma and Farydak (panobinostat) for multiple myeloma. Other HDAC inhibitors continue to be evaluated in preclinical studies and clinical trials [9]. HDAC inhibitors have been shown to induce tumor cell death, promote differentiation, suppress cell proliferation and cell cycle progression in vitro and inhibit angiogenesis, reduce tumor growth, and enhance immune responses of the host in vivo [8].

AR-42 (formerly known as OSU-HDAC42; Arno Therapeutics, Inc., Flemington, NJ) is a novel phenylbutyrate-based class I/IIB HDAC inhibitor originally [10] developed by our group and currently in clinical trials for hematologic malignancies. In preclinical studies, AR-42 was evaluated in vitro and in vivo in models of human prostate cancer [11], ovarian [12] and hepatocellular carcinoma [13], myeloma [14] and other B-cell malignancies [15], and a variety of canine cancers [16, 17]. For the most part, these studies have focused on antitumor activity in carcinomas and hematopoietic cancers. In this study, we investigate the antitumor effects of AR-42 in OS, an aggressive, highly malignant bone tumor of mesenchymal origin and the most common primary bone tumor of children and dogs. These data show that AR-42 is a potent inhibitor of OS cell viability and induces apoptosis. Furthermore, we show that AR-42 induces cell death via the activation of the intrinsic mitochondrial pathway through activation of caspase 3/7. This potent apoptotic activity is associated with the down regulation of survival pathways including Akt signaling and expression of survivin and Bcl-xl. In general, these effects of AR-42 were achieved with greater potency compared to suberoylanilide hydroxamic acid (SAHA; aka vorinostat), another pan-HDAC inhibitor. These results indicate that HDAC inhibitors may have therapeutic potential against both human and canine OS.

## Methods

### Reagents

The HDAC inhibitors AR-42 and SAHA were synthesized as described [10]**.** Stock solutions AR-42 and SAHA were prepared in DMSO and diluted in the indicated culture medium for treatment of cells in vitro. Antibodies against Akt, pAkt-Ser473, phosphor-glycogen synthase kinase-3 (GSK3)β, caspase-3, Bcl-xl, α-tubulin, cyclin D1, phosphor-p70 ribosomal protein S6 kinase (p70S6K), p-mTOR and PTEN were purchased from Cell Signaling Technologies (Beverly, MA). Additional polyclonal rabbit antibodies used were acetylated histones H3 (N-terminus) and H4 (Lys5/8/12/16) (Upstate Biotechnology, Inc., Lake Placid, NY), β-actin (Sigma, St. Louis, MO), and survivin (Novus Biologicals, Littleton, CO).

### Cell lines and cell culture

Canine OS cell lines, OSCA-2, −7.2, −16, −36, −39.1, −40, −50, were previously established in the laboratory of one of the authors (JFM). Normal canine osteoblasts were obtained from Cell Applications, Inc. (San Diego, CA). The canine OS cell line D17 and human OS cell lines SAOS-2, SJSA and U2OS were obtained from American Type Culture Collection (Manassas, VA). Cells were cultured in Dulbecco’s Modified Eagle Medium (DMEM) (OSCA-2, −16, −36, −39.1, −40, −50), RPMI-1640 (D17, OSCA-7.2), or McCoy’s medium (Gibco, Invitrogen, Carlsbad, CA) (SJSA, SAOS-2 and U2OS) supplemented with 10% fetal bovine serum (FBS, HyClone, Gemini, West Sacramento, CA) and antibiotics (100 U/ml penicillin, 0.1 mg/ml streptomycin) (Gibco) in a humidified incubator containing 5% CO_2_ at 37 °C.

### Cell viability assays

The effect of AR-42 and SAHA on the viability of canine and human tumor cells was assessed by the 3-(4,5-dimethylthiazol-2-yl)-2,5-diphenyltetrazolium bromide (MTT) assay (Sigma) as described previously [18]. Briefly, cells were seeded in 96-well plates at ~2500 cells per well in medium supplemented with 10% FBS. After 24 h, the cells were treated with varying concentrations (1–10 μM) of AR-42 and SAHA, dissolved in DMSO (final DMSO concentration ≤ 0.1%) for 24, 48 and 72 h. Controls were treated with DMSO vehicle alone at a concentration equal to that of drug-treated cells. After drug treatment, 22 μl of MTT reagent (5 mg/ml) was added to each well and the cells were incubated for up to 2 to 4 h at 37 °C. The absorbance was read on a plate reader (UV Spectromax M2 plate reader, Molecular Devices, Sunnyvale, CA) at 570 nm. The concentration of AR-42 and SAHA that inhibited cell viability by 50% (IC_50_) was determined using CompuSyn software (v. 3.0.1, ComboSyn, Inc., Paramus, NJ) and the values expressed as mean ± SD. All treatments were evaluated in triplicate in at least three independent experiments.

### Cell cycle analysis

Cells were exposed to AR-42 or SAHA at 1 and 10 μM concentrations for 48 h, washed with phosphate-buffered saline (PBS), resuspended in 500 μl of cold PBS, and then added drop wise to 70% ethanol and stored at 4 °C overnight. Cells were then washed twice in PBS and suspended in 500 μl of PBS containing 10 ug/ml of RNase A (LC, Laboratories, Woburn, CA) and 50 ug/ml of propidium iodide (Sigma) and assessed by BD FACS Calibur (Becton-Dickinson, San Jose, CA). Data were analyzed by Cell Quest flow software (Becton-Dickinson). A maximum of 10,000 cells within the gated region were analyzed for each treated and untreated sample. Experiments were replicated three times.

### Cell death detection ELISA

Drug-induced apoptotic cell death was determined by detection of DNA fragmentation using the Cell Death Detection ELISA kit (Roche, Indianapolis, IN). The ELISA was performed according to the manufacturer’s protocol and is based on the quantitative determination of cytoplasmic histone-associated DNA fragments in the form of mononucleosomes or oligonucleosomes generated after induced apoptotic death. Briefly, 3 × 10^5^ cells were cultured in RPMI/McCoy’s medium supplemented with 10% FBS in 100 mm tissue culture dishes for 24 h before treatment. Cells were treated with varying concentrations of AR-42 and SAHA (1–10 μM) and DMSO vehicle as control for 48 h. Approximately 100,000 cells were used per assay. The absorbance was read on a plate reader (UV Spectromax M2 plate reader, Molecular Devices) at a wavelength of 405–490 nm.

### Measurement of caspase 3/7 activity

Activation of the caspase 3/7 pathway following the drug treatment was measured by Sensolyte™ Homogeneous AMC Caspase-3/7 Assay kit (AnaSpec, San Jose, CA) following the manufacturer’s protocol. Briefly, 1x10^5^ cells per well were seeded in six-well plates and treated for 48 h at concentrations of 1 and 10 μM AR-42 or SAHA. Each treatment was performed in triplicate. After treatment for the indicated time, cells were lysed with lysis buffer to a final volume of 150 μl/well and 50 μl of caspase 3/7 substrate reagent was then added to the wells and incubated on a plate shaker for 30–60 s at 100–200 rpm. Fluorescence were read on a plate reader (UV Spectromax M2 plate reader, Molecular Devices) at an Ex/Em = 354 nm/442 nm and recorded after 1 h.

### Immunoblotting

For immunoblotting analysis, drug-treated (AR-42 and SAHA at 1 and 10 μM) and vehicle (DMSO)-treated cells were collected 48 h after treatment, washed in PBS, and lysed in M-PER protein extraction reagent (Pierce Biotechnology, Rockford, IL) unless otherwise stated. After centrifugation at 14,000 rpm for 15 min equal amounts of total protein from the cell lysates were resolved on 4–20% denaturing polyacrylamide gels (Invitrogen) and transferred to nitrocellulose membranes (PALL-Germany). After blocking with TBST containing 5% non-fat dry milk (Blotto, BioRad Laboratories, Hercules, CA) for 1 h, the membranes were incubated with indicated primary antibodies at 4 °C overnight and then washed three times with TBST. The membranes were probed with horseradish peroxidase conjugated secondary antibodies (Jackson Immune Research Laboratories, West Grove, PA) for 1 h at room temperature and washed three times with TBST. The blots were then developed with Western Lightning reagents (Perkin-Elmer, Waltham, MA).

### Drug combination studies

The effect of combining AR-42 with doxorubicin on cancer cell viability was evaluated in U2OS and D17 cells using the fixed-ratio method. Cells were treated with AR-42 and doxorubicin individually and in combination. For combination treatment of U2OS cells, drugs were combined at a concentration ratio of 3.3:1 (AR42:doxorubicin) and 2-fold serial dilutions were performed to generate a series of solutions containing AR-42 and doxorubicin at concentrations ranging from 0.125 to 8 μM and 0.0375 to 2.4 μM, respectively. For treatment of D17 cells, the concentrations of AR-42 in the combination ranged from 0.625 to 40 μM, and that of doxorubicin ranged from 0.04125 to 2.64 μM to yield a fixed concentration ratio of 15.2:1 (AR-42:doxorubicin). After treatments, cell viability was determined by MTT assays as described above. Dose-effect data for individual drugs and their combinations were analyzed for synergistic effects using the median-effect method of Chou and Talalay [19] using CompuSyn software (v. 3.0.1, ComboSyn, Inc.). Combination index (CI) values were calculated to characterize the nature of the drug interaction as defined by Chou and Talalay: CI = 1, additivity; CI < 1, synergism; CI > 1, antagonism. The dose reduction index (DRI) is a measure of the extent to which the dose of a drug in a synergistic combination is reduced, compared with the dose of the same drug alone, to achieve a given effect level. The DRI value for each drug was also calculated.

### Statistical analysis

**Fig. 1:**
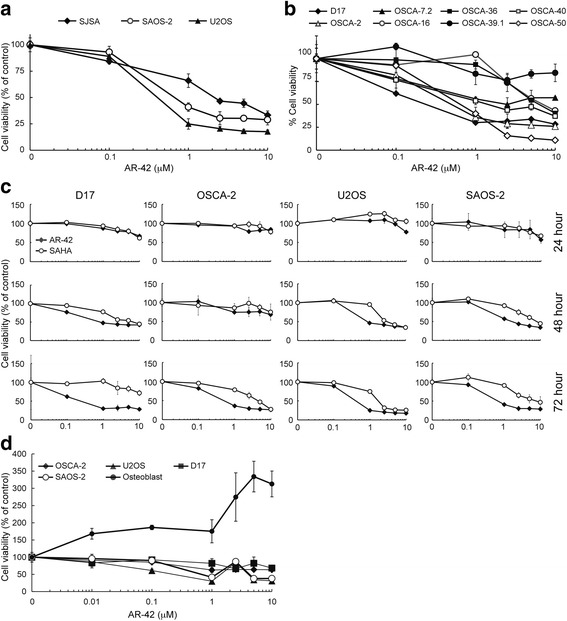
Antiproliferative effect of HDAC inhibitors AR-42 and SAHA on human and canine osteosarcoma cells. **a**, **b** Dose-dependent effects of AR-42 on cell viability after 72 h of treatment in human (SJSA, SAOS-2, U2OS) and canine (D17, OSCA-2, OSCA-7.2, OSCA-16, OSCA-40, OSCA-36, OSCA-39.1, OSCA-50,) OS cells. **c** Time- and dose-dependent effects of AR-42 and SAHA on cell viability in human (U2OS and SAOS-2) and canine (D17, OSCA-2) OS cells. **d** Dose-dependent effects of AR-42 on cell viability after 72 h of treatment in human (SAOS-2, U2OS) and canine (D17, OSCA-2) OS cells and normal canine osteoblasts. Cells were exposed to AR-42 or SAHA at the indicated concentrations for 24, 48 or 72 h and cell viability assessed by MTT assay. Data presented are the average of triplicate determinations from three independent experiments

Cell viability was measured in triplicate and averaged to achieve a single value for each combination of drug treatment, concentration, and cell line. The results were plotted over the three measurement periods and visually inspected (Fig. 1c). The cell viability at 72 h was compared by drug treatment assignment, AR-42 (*n* = 24) versus SAHA (*n* = 24). Samples were tested for normality (D ’Agostino-Pearson test) and equality of variance (Levene’s test). Inasmuch as samples were not normally distributed, a Mann-Whitney test for independent samples was performed to compare cell viability by drug treatment group. Analyses were performed using commercial software (MedCalc Statistical Software version 16.8, MedCalc Software bvba, Ostend, Belgium). Statistical significance was set at *P* < 0.05.

## Results

### HDAC inhibitors AR-42 and SAHA reduce viability of canine and human OS cells in a dose-dependent manner

The antiproliferative effects of AR-42 and SAHA were assessed in three human (SAOS-2, SJSA and U2OS) and eight canine (D17, OSCA-2, −7.2, −16, −36, −39.1, −40 and −50) OS cell lines. OS cells were treated with each drug over a concentration range of 0.1 to 10 μM and cell viability was measured by MTT assay at 24, 48 and 72 h of treatment. AR-42 caused dose-dependent reductions in cell viability in all of the human cell lines starting at 48 h of treatment, except in SJSA cells for which a decrease in viability was not seen until 72 h (Fig. 1a; 24 and 48 h data for SJSA are not shown). Similarly, AR-42 reduced cell viability in most of the canine cell lines, which, however, exhibited differential sensitivities ranging from near complete resistance (OSCA-39.1) to IC50 values of <1 μM at 72 h of treatment (D17, OSCA-2 and OSCA-50) (Fig. 1b). OSCA-16, −7.2 and −36 cell lines were relatively resistant. The IC50 values for sensitive human and canine OS cells ranged from 0.25 to 2.2 μM after 72 h of treatment. Comparison of the antiproliferative activities of AR-42 and SAHA was performed in sensitive human (U2OS, SAOS-2) and canine (D17, OSCA-2) OS cell lines which showed that both inhibitors induced time- and dose-dependent decreases in cell viability and that, in all the cell lines tested, AR-42 was more potent than SAHA (Fig. 1c). Finally, the sensitivity of non-malignant canine osteoblasts to AR-42 relative to human (SAOS-2, U2OS) and canine (OSCA-2, D17) OS cells was determined. As shown in Fig. 1d, the median percent cell viability at 72 h was significantly greater for SAHA-treated cells (*Z* = 2.474, *P* = 0.0133). This effect appeared to be present at all dilutions of the drugs and in all cell-lines, although the magnitude of the differences varied with combinations of drug concentration and cell lines.

### Reduced cell viability correlates with increased apoptosis

**Fig. 2:**
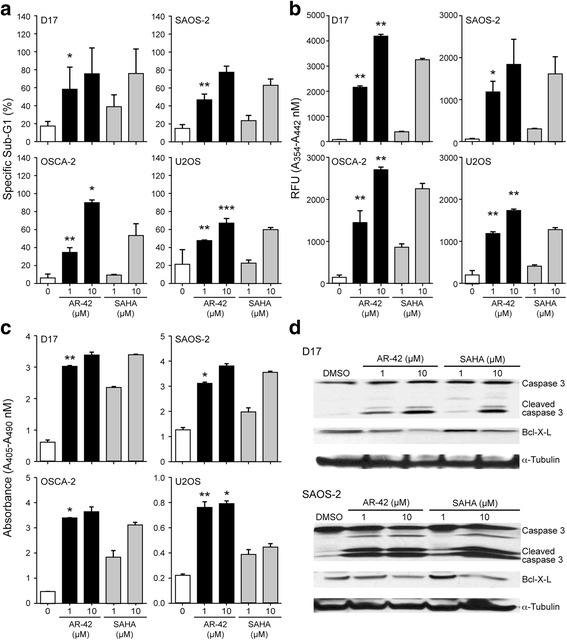
HDAC inhibitors AR-42 and SAHA induce apoptosis of osteosarcoma cells. **a** Dose-dependent increase of apoptosis in OS cells treated with AR-42 or SAHA as determined by analysis of cell cycle profiles by flow cytometry. Cells were exposed to DMSO, AR-42, or SAHA at the indicated concentrations for 48 h. The percentage of propidium iodide stained sub G1 cells is shown. Representative data from three independent experiments are presented. *p < 0.01, **p < 0.005, ***p < 0.0001. **b** Quantitation of caspase-3/7 enzymatic activity in OS cells treated with AR-42. OS cells were exposed to DMSO, AR-42, or SAHA at the indicated concentrations for 48 h. Caspase-3/7 enzymatic activity was determined using the SensoLyte® Homogeneous AMC Caspase-3 Assay Kit. *p < 0.01, **p < 0.005. **c** Dose-dependent effect of AR-42 on fragmented nucleosome accumulation in treated OS cells. OS cells were exposed to DMSO, AR-42, or SAHA at the indicated concentrations for 48 h. Fragmented cytoplasmic nucleosomal DNA was detected by Cell Death Detection ELISA Kit. *p < 0.001, **p < 0.0001. **d** Western blot analysis of apoptotic markers. OS cells were exposed to AR-42 or SAHA at the indicated concentrations for 48 h. Protein lysates were generated and separated by SDS-PAGE and Western blotting for caspase-3, Bcl-xL, and α-tubulin was performed

The antitumor effects of HDAC inhibitors have been linked to their ability to inhibit growth and induce apoptosis in cancer cells. To determine the ability of AR-42 to induce apoptosis in OS cells, we analyzed the effects of AR-42 and SAHA treatment on OS cells by cell cycle analysis, quantitation of nucleosome fragmentation, and measurement of caspase activity. Both inhibitors induced dose-dependent apoptosis as indicated by an increased sub-G1 cell population after 48 h of drug treatment, which inversely correlated with a reduced percentage of cycling cells, confirming decreased cell proliferation (Fig. 2a). Relative to SAHA, AR-42-treated cells exhibited a significantly greater increase in apoptosis at the 1 μM concentration in all cell lines tested (Fig. 2a). Increases in nucleosome fragmentation paralleled those in the subG1 cell population (Fig. 2b). Furthermore, both HDAC inhibitors increased caspase 3/7 enzymatic activity in all cell lines, consistent with activation of the intrinsic apoptotic pathway (Fig. 2c). Lastly, Western blotting demonstrated a dose-dependent increase in caspase 3 cleavage and a decrease in Bcl-xl expression following HDAC inhibition (Fig. 2d). These results demonstrate that AR-42 induces apoptosis in both canine and human OS cell lines at low micromolar drug concentrations.

### HDAC inhibitors increased acetylation of histone H3 and H4 in OS cells

**Fig. 3:**
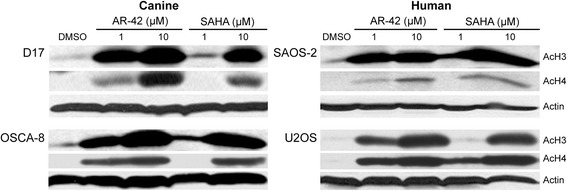
AR-42 and SAHA treatment enhanced histone acetylation in both human and canine osteosarcoma cells. OS cells were treated with DMSO and the indicated concentrations of AR-42 or SAHA for 48 h. The acetylation status of histones H3 and H4 were determined by Western blotting. β-actin was used as a loading control

The antitumor effects of HDAC inhibitors are often the consequence of altered gene transcription resulting from increased histone acetylation. To examine the relationship between HDAC inhibition and histone acetylation in OS cells we assessed the levels of acetylated histones in OS cells following treatment with AR-42 or SAHA. Western blot analysis after 48 h of treatment revealed a dose-dependent induction of acetylated histones H3 and H4 in both human and canine OS cell lines (Fig. 3) demonstrating that these drugs actively modulate chromatin by inhibiting HDAC activity in the tumor cell lines. These effects on histone acetylation correlated with the differential and dose-dependent effects of AR-42 and SAHA on cell viability and apoptosis.

### Potent reduction in cell survival/viability is associated with attenuation of AKT activation and downstream signaling molecules

**Fig. 4:**
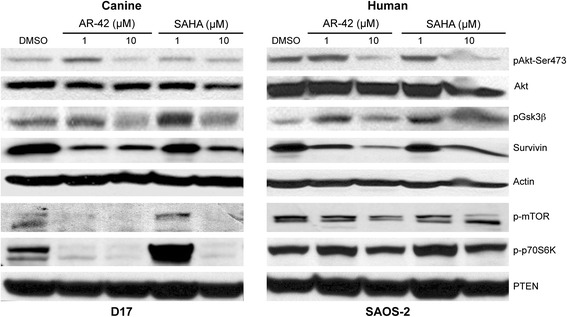
HDAC inhibition causes dose-dependent decreases in Akt phosphorylation and multiple downstream signaling molecules**.** D17 and SAOS-2 OS cells were exposed to the indicated concentrations of AR-42, SAHA, and DMSO for 48 h. Protein lysates were generated and separated by SDS-PAGE and Western blotting was performed with the indicated antibodies. Representative immunoblots showing dose-dependent effects of AR-42 and SAHA on Akt and GSK3β phosphorylation and survivin. β-Actin was used as a loading control. Protein lysates of similarly treated cells were immunoblotted with mTOR and p70S6K phospho-antibodies

Both AR-42 and, to a lesser extent, SAHA have been shown to decrease Akt phosphorylation in prostate cancer cells [20] attributable to the disruption of HDAC-protein phosphatase-1 (PP1) complexes leading to increased PP1-Akt association [21]. Thus, we investigated the effects of both drugs on Akt phosphorylation and downstream markers of Akt signaling. Western blot analysis revealed that, after 48 h of treatment with 10 μM of either AR-42 or SAHA, pAkt-Ser^473^ was reduced in 2 of 3 human and 2 of 4 canine OS cell lines (data for D17 and SAOS-2 cells are shown, Fig. 4). Similarly, decreases in the phosphorylation of GSK3β, a direct phosphorylation target of Akt, were also observed in cells treated with higher concentrations of drug, which confirmed the functional suppression of Akt signaling in these HDAC inhibitor-treated cells. HDAC inhibitor treatment also caused reductions in the phosphorylation of mTOR and its substrate, p70S6K, more prominently in D17 cells than in SAOS-2 cells, in which the effects were small. The mTOR pathway is another downstream effector of Akt signaling and its dysregulation has been implicated in OS. At 1 μM AR-42 and SAHA, effects on Akt signaling were inconsistent, ranging from minor reductions to apparent lack of effect or even increases in these signaling markers, which was unexpected in light of clear effects of this concentration of drug on cell viability, apoptosis, and/or histone acetylation. Phosphatase and tensin homolog (PTEN) negatively regulates Akt signaling by antagonizing the action of phosphatidylinositol 3–kinase (PI3K) [22]. Thus, the effect of AR-42 on the expression of PTEN was also assessed. Neither AR-42 nor SAHA altered the abundance of PTEN protein in OS cell lines, suggesting that PTEN was not involved in the HDAC inhibitor-mediated suppression of pAkt-Ser^473^.

The up-regulation of survivin, an inhibitor of apoptosis protein (IAP) family member, has been reported to be associated with poor prognosis and increased risk of metastasis in OS [23–25]. Previous studies have demonstrated that HDAC inhibitors, including AR-42, can suppress survivin expression in cancer cells [13, 20, 26]. Moreover, survivin expression has been shown to be regulated by the Akt/p70S6K1 pathway [27]. As shown in Fig. 4, treatment with either HDAC inhibitor resulted in a dose-dependent reduction of survivin in both human and canine OS cells. These data suggest that AR-42 induces apoptosis of OS tumor cell lines through modulation of key cellular proteins.

### AR-42 enhances the cytotoxic effects of doxorubicin in OS cells

**Fig. 5:**
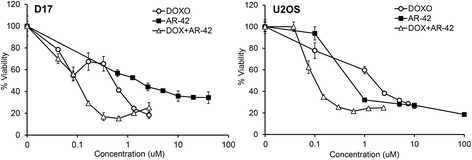
The combination of AR-42 and doxorubicin have synergistic activity in osteosarcoma cells. Dose-response curves for D17 and U2OS cells treated with AR-42 and doxorubicin alone and in combination. The concentrations plotted for the combination are those of doxorubicin. D17 and U2OS cells were treated for 72 h with various concentrations of either AR-42 or doxorubicin alone, or in combination, with a fixed ratio of AR-42 to doxorubicin. Cell viability was assessed by MTT assay. Data presented is the average of triplicate determinations from two independent experiments

Several preclinical studies have described synergistic effects of HDAC inhibitors in combination with a diverse range of cytotoxic and targeted therapeutic agents [5]. AR-42 itself has been reported to sensitize prostate cancer and hepatocellular carcinoma cells to DNA double-strand break-inducing chemotherapeutic agents [28] and radiotherapy [29], respectively. Consequently, we investigated the potential synergistic activity of the combination of AR-42 and doxorubicin in human and canine OS cells. U2OS and D17 cells were treated with AR-42 and doxorubicin over a range of doses both as single agents and in combinations containing the drugs at fixed concentration ratios. The dose-effect data generated after single agent and combination treatments of each cell line (Fig. 5) were subjected to median-effects analysis from which CI (combination index) values were derived to define the drug interactions. As shown in Table 1, for both cell lines the AR-42/doxorubicin combination was synergistic, as all CI values were less than 1 at dose levels that reduced cell viability by 50% (effective dose-50, ED50) or greater. Calculation of the dose reduction index (DRI) values revealed that, as a result of the synergism between AR-42 and doxorubicin, their IC50 values could be reduced by 2.06-fold and 4.03-fold, respectively, in U2OS cells, and 1.40-fold and 4.57-fold, respectively, in D17 cells (Table 2).

**Table 1:** Combination index (CI) values of AR-42 and doxorubicin at the indicated effective dose (ED) in osteosarcoma cell lines

Cell Line	ED50	ED75	ED90	ED95
D17	0.93	0.38	0.46	0.59
U2OS	0.74	0.23	0.07	0.03

**Table 2:** Dose reduction index (DRI)^a^ of AR-42 and doxorubicin in osteosarcoma cell lines

U2OS	AR-42	Doxorubicin
Single agent IC50 [μM]	2.55	1.45
Combination IC50 [μM]	1.24	0.36
DRI	2.06	4.03
D17	AR-42	Doxorubicin
Single agent IC50 [μM]	1.46	0.32
Combination IC50 [μM]	1.04	0.07
DRI	1.40	4.57

^a^Dose reduction index (DRI) indicates the extent to which the concentration of a drug can be reduced in the combination to achieve an effect level similar to that achieved as a single agent

## Discussion

Although strategies for treating primary OS tumors are generally effective, the development of metastasis, primarily to lung, remains the most common cause of mortality for patients. No significant improvements in outcomes for patients have occurred since the incorporation of neoadjuvant and adjuvant chemotherapy over 30 years ago [1]. In a recent review of drug development strategies for OS, it was emphasized that new therapeutic targets are needed and that, where feasible, the inclusion of the dog with naturally occurring OS is of great value for defining the activity of new drugs [30]. In this study, we used a comparative in vitro approach to investigate the antitumor effects of a novel HDAC inhibitor, AR-42, in human and canine OS cells.

DNA methylation and histone acetylation are important epigenetic mechanisms that regulate gene transcription in cells. HDAC inhibitors modulate chromatin structure through hyperacetylation of histones that critically affect transcription of various genes, including tumor suppressors, and mediate their effects in a cell type dependent manner [5, 7]. In cancer, HDAC inhibitors mediate changes in expression of genes involved in regulation of cell survival, growth, check point control, apoptosis, differentiation, migration and angiogenesis. In general, cancer cells appear to be more sensitive than normal cells to the effects of HDAC inhibitors, suggesting great potential for the development and therapeutic use of these agents to treat cancer [5, 7].

AR-42 is a novel hydroxamate-tethered phenylbutyrate derivative that inhibits both class-I and -II deacetylases; i.e., it is a pan-HDAC inhibitor. Previously, we and others reported that AR-42 significantly reduced cell viability and proliferation and increased apoptosis in a variety of human cancer cell lines, in vitro and in vivo; primarily in carcinomas (e.g. prostate [11], hepatocellular [13], ovarian [12] and hematopoietic cancers (e.g. myeloma [14], chronic lymphocytic leukemia [15]). We also have shown that AR-42 has antitumor effects in canine cancer cells [16], including malignant mast cells, where we showed that the antitumor effects of AR-42 are mediated via down-regulation of constitutively activated *KIT* [17]. In the former study, in addition to demonstrating the antiproliferative effects of AR-42 in canine carcinomas and malignant hematopoietic cells, similar effects were observed in a single OS cell line. In this study we further evaluated the effects of AR-42 in both human and canine OS cell lines. Spontaneous OS in people and dogs share common clinical, morphological, genetic, and transcriptional profile characteristics, making OS in the dog an excellent large animal preclinical model for drug development [4].

The concentration range of AR-42 used for testing (up to 10 μM) was selected based on previously published data on AR-42’s activity in a variety of cancer cell types and on the contention that relevant tissue concentrations of >10 μM were unlikely to be achieved in vivo. In support of this view, newly published pharmacokinetic data on AR-42 showed good penetration in bone marrow (6 μM) in leukemic mice following oral dosing of 40 mg/kg thrice weekly for 2.5 weeks (Cheng et al., AAPS J, 18:737–45, 2016). In this study, both human and canine OS cells showed greater sensitivity to treatment with HDAC inhibitors compared to normal canine osteoblasts, suggesting tumor cell specific anti-apoptotic effects of HDAC inhibition. The lower sensitivities of nonmalignant cells relative to the corresponding malignant cell types to the effects of AR-42 have been reported for various types of cells, including prostate epithelial cells (20), oral keratinocytes (Bai et al., Oral Oncol, 47:1127, 2011), ovarian surface epithelial cells (12), and hepatocytes (13). As anticipated, AR-42 increased histone acetylation in all OS cell lines, although the extent to which this occurred varied between cell lines. In all sensitive cell lines, AR-42 significantly inhibited cell viability and induced apoptosis at lower concentrations than SAHA. Decreases in cell viability correlated with an increase in apoptotic activity, as evidenced by an increase in cleaved caspase 3 protein, increased caspase 3/7 enzymatic activity, cytoplasmic accumulation of fragmented nucleosomes, and an increase in the subG1 cell population. Several other HDAC inhibitors, including trichostatin A (TSA) [31], SAHA [31], FR901228 [32], and MS-275 [33] have been shown to induce histone hyperacetylation and decrease cell viability in human OS cell lines.

Our results suggest that HDAC inhibitors have pleiotropic effects on OS cells in vitro, including increased acetylation of histones, inhibition of Akt activity with consequent effects on downstream effectors of Akt signaling, including GSK3β, mTOR, and survivin, suppression of anti-apoptotic Bcl-xl expression, and activation of intrinsic mechanisms of apoptosis in a dose-dependent manner. These observations suggest that the potent antitumor activity of HDAC inhibitors is due to the ability to activate multiple antitumor mechanisms including increased histone acetylation inducing increased gene transcription, inhibition of cell survival and growth through inhibition of Akt signaling, and increased induction of apoptosis via the intrinsic pathway. Surprisingly, the observed effects of the low dose (1 μM) of AR-42 and SAHA on Akt signaling markers (Fig. 4) were inconsistent with their effects on cell viability, apoptosis and histone acetylation. Perhaps, these data suggest that, under these conditions, Akt signaling is not a major mediator of HDAC inhibitor-induced apoptosis in these cell types. Indeed, multiple pro-apoptotic mechanisms in cancer cells have been implicated in the anticancer effects of HDAC inhibition, including both extrinsic and intrinsic apoptotic pathways, cell cycle arrest, ROS production, and transcriptional induction of pro-apoptotic *BCL2* family genes [34, 35].

Recently, the aggressiveness of OS was linked to specific gene signatures that are due in part to modulation of the epigenetic landscape by RB. These signatures could be reversed to resemble less aggressive OS or normal bone by HDAC and DNA methyltransferase (DNMT) inhibition with SAHA and Zebularine [36]. This may explain why more aggressive tumors are more sensitive to HDAC inhibition [37].

Importantly, the concentrations of AR-42 required to induce histone acetylation, decrease cell proliferation, and induce apoptosis occurred at low micromolar concentrations that are biologically relevant and correlated with the inhibitory concentrations tested in other cancer models. Furthermore, the combination of AR-42 and doxorubicin resulted in a significant decrease in cell viability compared to treatment with either agent alone, suggesting synergistic effects of the drug combination and a potential clinical use for OS therapy. Similarly, although the weak HDAC inhibitor valproic acid did not decrease OS cell viability at physiologically relevant concentrations, it did sensitize human and canine OS cells to doxorubicin [38] and was well tolerated in combination with doxorubicin in dogs with spontaneously occurring solid tumors [39].

## Conclusions

These data demonstrate that HDAC inhibitors induced apoptosis and sensitized both human and canine OS cells to cytotoxic chemotherapy. AR-42 was more potent than SAHA as it reduced the viability of canine and human OS cells at lower concentrations. Furthermore, AR-42 enhanced the cytotoxic effects of doxorubicin, a drug that is currently used for treatment of OS clinically. These synergistic interactions can be further explored for the treatment of OS in humans. These results further validate the comparative oncology approach to drug development for OS [40].

## Abbreviations

DMSO: Dimethyl sulfoxide
DNMT: DNA methyltransferase
DRI: Dose reduction index
ELISA: Enzyme-linked immunosorbent assay
FACS: Fluorescence activated cell sorter
FDA: Food and Drug Administration
GSK3: Glycogen synthase kinase-3
HAT: Histone acetyl transferase
HDAC: Histone deacetylase
IAP: Inhibitor of apoptosis protein
IC_50_: Inhibitory concentration 50%
MTT: 3-(4,5-dimethylthiazol-2-yl)-2,5-diphenyltetrazolium bromide
OS: Osteosarcoma
p70S6K: p70 ribosomal protein S6 kinase
RNA: Ribonucleic acid
SAHA: Suberoylanilide hydroxamic acid

## References

[1] Isakoff MS, Bielack SS, Meltzer P, Gorlick R (2015). Osteosarcoma: Current Treatment and a Collaborative Pathway to Success. J Clin Oncol.

[2] Rowell JL, McCarthy DO, Alvarez CE (2011). Dog models of naturally occurring cancer. Trends Mol Med.

[3] Mirabello L, Troisi RJ, Savage SA (2009). Osteosarcoma incidence and survival rates from 1973 to 2004: data from the Surveillance, Epidemiology, and End Results Program. Cancer.

[4] Fenger JM, London CA, Kisseberth WC (2014). Canine osteosarcoma: a naturally occurring disease to inform pediatric oncology. ILAR J.

[5] Marks PA, Xu WS (2009). Histone deacetylase inhibitors: Potential in cancer therapy. J Cell Biochem.

[6] Marks PA (2010). The clinical development of histone deacetylase inhibitors as targeted anticancer drugs. Expert Opin Investig Drugs.

[7] Barneda-Zahonero B, Parra M (2012). Histone deacetylases and cancer. Mol Oncol.

[8] Bose P, Dai Y, Grant S (2014). Histone deacetylase inhibitor (HDACI) mechanisms of action: emerging insights. Pharmacol Ther.

[9] Guha M (2015). HDAC inhibitors still need a home run, despite recent approval. Nat Rev Drug Discov.

[10] Lu Q, Wang DS, Chen CS, Hu YD, Chen CS (2005). Structure-based optimization of phenylbutyrate-derived histone deacetylase inhibitors. J Med Chem.

[11] Sargeant AM, Rengel RC, Kulp SK, Klein RD, Clinton SK, Wang YC, Chen CS (2008). OSU-HDAC42, a histone deacetylase inhibitor, blocks prostate tumor progression in the transgenic adenocarcinoma of the mouse prostate model. Cancer Res.

[12] Yang YT, Balch C, Kulp SK, Mand MR, Nephew KP, Chen CS (2009). A rationally designed histone deacetylase inhibitor with distinct antitumor activity against ovarian cancer. Neoplasia.

[13] Lu YS, Kashida Y, Kulp SK, Wang YC, Wang D, Hung JH, Tang M, Lin ZZ, Chen TJ, Cheng AL (2007). Efficacy of a novel histone deacetylase inhibitor in murine models of hepatocellular carcinoma. Hepatology.

[14] Zhang S, Suvannasankha A, Crean CD, White VL, Chen CS, Farag SS (2011). The novel histone deacetylase inhibitor, AR-42, inhibits gp130/Stat3 pathway and induces apoptosis and cell cycle arrest in multiple myeloma cells. Int J Cancer.

[15] Lucas DM, Alinari L, West DA, Davis ME, Edwards RB, Johnson AJ, Blum KA, Hofmeister CC, Freitas MA, Parthun MR (2010). The novel deacetylase inhibitor AR-42 demonstrates pre-clinical activity in B-cell malignancies in vitro and in vivo. PLoS One.

[16] Kisseberth WC, Murahari S, London CA, Kulp SK, Chen CS (2008). Evaluation of the effects of histone deacetylase inhibitors on cells from canine cancer cell lines. Am J Vet Res.

[17] Lin TY, Fenger J, Murahari S, Bear MD, Kulp SK, Wang D, Chen CS, Kisseberth WC, London CA (2010). AR-42, a novel HDAC inhibitor, exhibits biologic activity against malignant mast cell lines via down-regulation of constitutively activated Kit. Blood.

[18] Alvarez FJ, Murahari S, Couto CG, Rosol TJ, Kulp SK, Chen CS, Kisseberth WC (2007). 3-Phosphoinositide-dependent protein kinase-1/Akt signalling and inhibition in a canine prostate carcinoma cell line. Vet Comp Oncol.

[19] Chou TC, Talalay P (1984). Quantitative analysis of dose-effect relationships: the combined effects of multiple drugs or enzyme inhibitors. Adv Enzyme Regul.

[20] Kulp SK, Chen CS, Wang DS, Chen CY, Chen CS (2006). Antitumor effects of a novel phenylbutyrate-based histone deacetylase inhibitor, (S)-HDAC-42, in prostate cancer. Clin Cancer Res.

[21] Chen CS, Weng SC, Tseng PH, Lin HP, Chen CS (2005). Histone acetylation-independent effect of histone deacetylase inhibitors on Akt through the reshuffling of protein phosphatase 1 complexes. J Biol Chem.

[22] Chalhoub N, Baker SJ (2009). PTEN and the PI3-kinase pathway in cancer. Annu Rev Pathol.

[23] Wang W, Luo H, Wang A (2006). Expression of survivin and correlation with PCNA in osteosarcoma. J Surg Oncol.

[24] Trieb K, Lehner R, Stulnig T, Sulzbacher I, Shroyer KR (2003). Survivin expression in human osteosarcoma is a marker for survival. Eur J Surg Oncol.

[25] Osaka E, Suzuki T, Osaka S, Yoshida Y, Sugita H, Asami S, Tabata K, Sugitani M, Nemoto N, Ryu J (2007). Survivin expression levels as independent predictors of survival for osteosarcoma patients. J Orthop Res.

[26] Seo SK, Jin HO, Lee HC, Woo SH, Kim ES, Yoo DH, Lee SJ, An S, Rhee CH, Hong SI (2008). Combined effects of sulindac and suberoylanilide hydroxamic acid on apoptosis induction in human lung cancer cells. Mol Pharmacol.

[27] Zhao P, Meng Q, Liu LZ, You YP, Liu N, Jiang BH (2010). Regulation of survivin by PI3K/Akt/p70S6K1 pathway. Biochem Biophys Res Commun.

[28] Chen CS, Wang YC, Yang HC, Huang PH, Kulp SK, Yang CC, Lu YS, Matsuyama S, Chen CY, Chen CS (2007). Histone deacetylase inhibitors sensitize prostate cancer cells to agents that produce DNA double-strand breaks by targeting Ku70 acetylation. Cancer Res.

[29] Lu YS, Chou CH, Tzen KY, Gao M, Cheng AL, Kulp SK, Cheng JC (2012). Radiosensitizing effect of a phenylbutyrate-derived histone deacetylase inhibitor in hepatocellular carcinoma. Int J Radiat Oncol Biol Phys.

[30] Khanna C, Fan TM, Gorlick R, Helman LJ, Kleinerman ES, Adamson PC, Houghton PJ, Tap WD, Welch DR, Steeg PS (2014). Toward a drug development path that targets metastatic progression in osteosarcoma. Clin Cancer Res.

[31] Roh MS, Kim CW, Park BS, Kim GC, Jeong JH, Kwon HC, Suh DJ, Cho KH, Yee SB, Yoo YH (2004). Mechanism of histone deacetylase inhibitor Trichostatin A induced apoptosis in human osteosarcoma cells. Apoptosis.

[32] Watanabe K, Okamoto K, Yonehara S (2005). Sensitization of osteosarcoma cells to death receptor-mediated apoptosis by HDAC inhibitors through downregulation of cellular FLIP. Cell Death Differ.

[33] Rao-Bindal K, Koshkina NV, Stewart J, Kleinerman ES (2013). The histone deacetylase inhibitor, MS-275 (entinostat), downregulates c-FLIP, sensitizes osteosarcoma cells to FasL, and induces the regression of osteosarcoma lung metastases. Curr Cancer Drug Targets.

[34] Bolden JE, Shi W, Jankowski K, Kan CY, Cluse L, Martin BP, MacKenzie KL, Smyth GK, Johnstone RW (2013). HDAC inhibitors induce tumor-cell-selective pro-apoptotic transcriptional responses. Cell Death Dis.

[35] Frew AJ, Johnstone RW, Bolden JE (2009). Enhancing the apoptotic and therapeutic effects of HDAC inhibitors. Cancer Lett.

[36] Scott MC, Sarver AL, Tomiyasu H, Cornax I, Van Etten J, Varshney J, O’Sullivan MG, Subramanian S, Modiano JF (2015). Aberrant retinoblastoma (RB)-E2F transcriptional regulation defines molecular phenotypes of osteosarcoma. J Biol Chem.

[37] Thayanithy V, Park C, Sarver AL, Kartha RV, Korpela DM, Graef AJ, Steer CJ, Modiano JF, Subramanian S (2012). Combinatorial treatment of DNA and chromatin-modifying drugs cause cell death in human and canine osteosarcoma cell lines. PLoS One.

[38] Wittenburg LA, Bisson L, Rose BJ, Korch C, Thamm DH (2011). The histone deacetylase inhibitor valproic acid sensitizes human and canine osteosarcoma to doxorubicin. Cancer Chemother Pharmacol.

[39] Wittenburg LA, Gustafson DL, Thamm DH (2010). Phase I pharmacokinetic and pharmacodynamic evaluation of combined valproic acid/doxorubicin treatment in dogs with spontaneous cancer. Clin Cancer Res.

[40] Paoloni M, Khanna C (2008). Translation of new cancer treatments from pet dogs to humans. Nat Rev Cancer.

